# Efficacy of Zolpidem Tartrate in Benzodiazepine-Refractory Catatonia: A Case Report

**DOI:** 10.7759/cureus.104543

**Published:** 2026-03-02

**Authors:** Matthew B Urban, Matthew Garofalo

**Affiliations:** 1 Psychiatry, New York Medical College, Valhalla, USA; 2 Psychiatry, Westchester Medical Center, Valhalla, USA

**Keywords:** benzodiazepines, catatonia, refractory catatonia, schizophrenia, zolpidem, zolpidem challenge

## Abstract

This study aims to describe the clinical response to a zolpidem challenge in a patient with benzodiazepine-refractory catatonia and highlight its potential role as an alternative pharmacologic intervention when standard therapies are ineffective or contraindicated.

We report the case of a 25-year-old woman with schizophrenia and severe catatonia admitted to an inpatient psychiatric unit. Catatonic features included psychomotor slowing, mutism, negativism, staring, and stupor. The patient demonstrated no clinical improvement with benzodiazepine therapy and was unable to pursue electroconvulsive therapy (ECT).

A zolpidem challenge was initiated at 10 mg to assess symptomatic response. Clinical status was monitored through behavioral observation, functional engagement, and tolerability during dose titration and maintenance. Following zolpidem administration, the patient showed partial improvement in psychomotor slowing, mutism, and alertness, with increased verbal engagement and participation in unit activities after titration to divided daily dosing.

This case supports prior literature suggesting that zolpidem may provide short-term symptomatic benefit in benzodiazepine-refractory catatonia. The observed improvements in psychomotor slowing, mutism, and engagement were consistent with previously reported transient responses. However, subsequent sedation and diminishing therapeutic effect underscore limitations related to durability and tolerability with continued use.

When benzodiazepines are ineffective and ECT is contraindicated or unavailable, zolpidem may represent a reasonable alternative strategy. Further study is needed to clarify optimal dosing, durability of response, and safety.

## Introduction

Catatonia is a neuropsychiatric syndrome characterized by motor, behavioral, and autonomic abnormalities, including mutism, stupor, negativism, posturing, and psychomotor slowing. It occurs in association with primary psychiatric disorders, particularly mood and psychotic disorders, as well as medical and neurologic conditions [1,2]. Current models implicate dysfunction of cortico-striato-thalamo-cortical circuitry with impaired regulation of excitatory and inhibitory neurotransmission. Dysregulated GABAergic and glutamatergic signaling have also been described [3,4].

First-line treatment typically consists of benzodiazepines, most commonly lorazepam, with reported response rates ranging from 66% to 100% in acute cases [1]. However, up to 27% of patients show only partial symptom reduction with benzodiazepines [4]. When patients do not respond to benzodiazepines, electroconvulsive therapy (ECT) is recommended. Response rates of 80%-100% have been reported with ECT, including up to 60% in benzodiazepine-refractory cases [1,3]. Despite this, some patients have limited access to ECT or cannot tolerate standard therapies, creating a need for alternative treatment strategies. Other second-line options include NMDA receptor antagonists, such as amantadine and memantine [5].

Zolpidem, a non-benzodiazepine hypnotic, has emerged in case reports and small series as a potential intervention for benzodiazepine-refractory catatonia. It acts as a positive allosteric modulator of GABA-A receptors with selectivity for the α1 subunit. This likely mediates its sedative-hypnotic effects and may underlie its paradoxical therapeutic benefit in the setting of proposed GABAergic dysfunction [6]. Reports describe rapid, but often transient improvement following administration, commonly referred to as a “zolpidem challenge” [7-10]. A recent systematic review identified 35 published cases with an overall response rate of approximately 80%, with outcomes varying by use as a challenge agent, monotherapy, or augmentation strategy [8]. The American Psychiatric Association Resource Document on Catatonia recognizes zolpidem as a promising alternative in refractory cases, while emphasizing that current evidence remains limited and largely case based [1].

We present a case of benzodiazepine-refractory catatonia demonstrating partial but time-limited response to scheduled zolpidem therapy, highlighting both potential benefits and important tolerability limitations.

## Case presentation

A 25-year-old woman with schizophrenia, multiple psychiatric inpatient hospitalizations, and previous suicide attempts was admitted for inpatient psychiatric care at Westchester Medical Center for severe psychosis and catatonia. On presentation, she demonstrated severe psychosis, hallucinations, and dysphoria. She also exhibited classic catatonic features, including psychomotor slowing, mutism, negativism, staring, and stupor, with marked functional impairment and limited verbal engagement. The pharmacologic regimen at admission included clozapine 75 mg daily, benztropine 0.5 mg twice daily, metformin 500 mg daily, duloxetine 60 mg daily, fludrocortisone 0.2 mg nightly, and atenolol 12.5 mg daily.

An initial trial of benzodiazepine therapy with clonazepam (0.5 mg each morning and 1 mg nightly) resulted in no meaningful clinical improvement and was associated with worsening sedation, increased isolation, and prolonged time spent in bed. Due to the lack of response and poor tolerability, clonazepam was reduced and discontinued. The patient declined ECT and did not meet criteria for involuntary ECT under state regulations; therefore, alternative pharmacologic strategies were pursued.

Given the limited response to benzodiazepines and the inability to pursue ECT, a zolpidem challenge was initiated as an alternative therapeutic strategy. Zolpidem was initiated at 10 mg to assess for clinical response. Clinical status was monitored through serial behavioral observation, functional engagement on the unit, verbal output, and medication tolerability during dose titration and maintenance. The intervention was performed in an inpatient setting with close monitoring for sedation and respiratory compromise.

Within 24 hours of the initial 10 mg zolpidem challenge, mild improvement was observed, including reduced psychomotor slowing, decreased duration of stupor, and increased spontaneous verbal output. Negativism and staring behaviors persisted. Over the subsequent several days, the dose was titrated to 10 mg twice daily in response to partial improvement. By day 7 of zolpidem therapy, the regimen was modified to 5 mg three times daily to reduce sedation while attempting to maintain therapeutic benefit. The patient became less sedated and more interactive, participating in group activities and milieu programming, ambulating more frequently on the unit, and demonstrating increased spontaneous speech. Negativism improved slightly, and staring episodes became shorter and less frequent.

After approximately two weeks of scheduled therapy, the patient developed progressively increasing sedation accompanied by diminishing therapeutic response. Zolpidem was gradually tapered and ultimately discontinued after 25 days of treatment due to intolerable sedation and reduced benefit.

Informed consent for publication was obtained from the patient. Institutional review board exemption was granted.

## Discussion

This case contributes to the growing body of literature suggesting that zolpidem may offer transient symptomatic benefit in select patients with benzodiazepine-refractory catatonia [7,8,10]. Zolpidem administration was associated with improvements in psychomotor slowing, mutism, alertness, and engagement in unit activities, allowing for greater participation in daily care and therapeutic programming. Although clinical improvement was observed, a standardized rating scale, such as the Bush-Francis Catatonia Rating Scale, was not systematically documented throughout hospitalization, limiting objective quantification of treatment response. Retrospective scoring was not feasible due to the narrative nature of daily documentation, which further limits reproducibility and comparison with other published cases. Additionally, concurrent psychotropic medications and the natural fluctuation of catatonia may have influenced the clinical course.

The partial and time-limited response observed in this patient, along with the development of sedation and apparent tachyphylaxis, highlights important limitations of zolpidem therapy. While initial improvements in psychomotor slowing, mutism, and engagement were clinically meaningful, durability was limited and adverse effects ultimately necessitated discontinuation. These findings underscore the need for careful dose titration, close monitoring for sedation, and realistic expectations regarding treatment sustainability when zolpidem is used off-label for catatonia [1,8]. Prior reports similarly describe a rapid onset of improvement with variable durability, often requiring repeated dosing and careful monitoring [7-9].

Beyond zolpidem, this case supports broader investigation into pharmacologic agents that target related neurobiological mechanisms [1,3,4]. Compounds that modulate specific GABA-A receptor subunits, glutamatergic signaling, or thalamocortical connectivity may represent potential therapeutic avenues for refractory catatonia and other treatment-resistant neuropsychiatric syndromes [1,4,6]. Systematic research into these mechanistic pathways may facilitate the development of more precise and durable interventions while minimizing sedative burden [1,5].

Dissemination of individual clinical experiences remains valuable in rare or refractory conditions where randomized data are limited. Case reports can generate hypotheses, inform clinical decision-making, and expand awareness of potential therapeutic options for patients who have exhausted standard treatments. This is particularly relevant in catatonia, where treatment response can be heterogeneous and individualized [1,2,4]. Even partial symptomatic relief in patients with severe catatonia may meaningfully improve daily functioning, safety, and quality of life.

Future case reports and prospective investigations would benefit from the systematic use of validated instruments, such as the Bush-Francis Catatonia Rating Scale, to allow objective measurement of symptom severity, treatment response, and durability over time.

## Conclusions

Although limited to a single patient, this case illustrates that zolpidem was associated with partial and time-limited symptomatic improvement in a patient with benzodiazepine-refractory catatonia who was unable to pursue ECT. The observed benefits in psychomotor activity, mutism, and engagement were clinically meaningful but not sustained, as increasing sedation and apparent tachyphylaxis ultimately limited continued use. While zolpidem’s selective agonism at the α1 subunit of the GABA-A receptor may contribute to its effect, catatonia remains a multifaceted pathophysiologic process that requires individualized treatment and careful clinical monitoring.

Further systematic study is needed to clarify optimal dosing, durability of response, and safety over time. Investigation into alternative pharmacologic approaches targeting related neurobiological mechanisms may expand treatment options for refractory cases. Even modest functional improvement can meaningfully enhance patient safety and day-to-day engagement in severe catatonia.
